# OxMaR: Open Source Free Software for Online Minimization and Randomization for Clinical Trials

**DOI:** 10.1371/journal.pone.0110761

**Published:** 2014-10-29

**Authors:** Christopher A. O’Callaghan

**Affiliations:** Nuffield Department of Clinical Medicine, University of Oxford, Oxford, United Kingdom; University of Oxford, United Kingdom

## Abstract

Minimization is a valuable method for allocating participants between the control and experimental arms of clinical studies. The use of minimization reduces differences that might arise by chance between the study arms in the distribution of patient characteristics such as gender, ethnicity and age. However, unlike randomization, minimization requires real time assessment of each new participant with respect to the preceding distribution of relevant participant characteristics within the different arms of the study. For multi-site studies, this necessitates centralized computational analysis that is shared between all study locations. Unfortunately, there is no suitable freely available open source or free software that can be used for this purpose. OxMaR was developed to enable researchers in any location to use minimization for patient allocation and to access the minimization algorithm using any device that can connect to the internet such as a desktop computer, tablet or mobile phone. The software is complete in itself and requires no special packages or libraries to be installed. It is simple to set up and run over the internet using online facilities which are very low cost or even free to the user. Importantly, it provides real time information on allocation to the study lead or administrator and generates real time distributed backups with each allocation. OxMaR can readily be modified and customised and can also be used for standard randomization. It has been extensively tested and has been used successfully in a low budget multi-centre study. Hitherto, the logistical difficulties involved in minimization have precluded its use in many small studies and this software should allow more widespread use of minimization which should lead to studies with better matched control and experimental arms. OxMaR should be particularly valuable in low resource settings.

## Introduction

The use of randomization in clinical trials is well established and has become the standard mode of allocating participants to groups within a trial. In principle, randomization will allocate participants with particular characteristics, such as ethnicity or gender, equally to the different arms of the study, typically, to a control or an experimental arm. However, when the study size is relatively small, allocation to each arm of the study may fail to reach equality using simple randomization. Any inequalities in the characteristics of the different study groups, such as age, gender or ethnicity may affect the study outcome.

With very large studies the probability of inequality between study arms with randomization is substantially diminished, but for smaller studies several different approaches have been developed to address this potential problem. Block randomization can be used to perform randomization within subsets or blocks of participants, thus ensuring that there is reasonable equality, at least in the size of the allocation groups. However, with small block sizes it may be possible to predict the likely allocation of some participants unless rigorous blinding procedures are in place. Stratified randomization involves separate randomization for each defined stratum or subset of participants in a study to promote an equitable distribution of participant characteristics between the allocation groups. Stratified randomization requires some form of blocking within strata analogous to block randomization.

The procedure of minimization was developed as a method for reducing the imbalance between allocation groups with respect to a number of participant variables, such as age, gender or ethnicity [1], [2]. Although the first participant is randomized, subsequent participants are allocated using an algorithm that seeks to minimize imbalance between the groups. The underlying principle of minimization is that the allocation algorithm takes into consideration the characteristics of previously allocated participants. An optional random component can be incorporated into the allocation protocol so that allocation is weighted rather than absolutely determined by previous allocations. Minimization is a powerful way to reduce differences between allocation groups and is especially useful in small studies.

Minimization was first reported in 1974 [1] and a general approach was developed by Pocock and Simon, which included the possibility of weighted randomization [2]. Variations on the algorithms have been explored, but the underlying principle of minimizing differences between study groups is retained [3], [4]. The CONSORT (Consolidated Standards of Reporting Trials) group endorses the use of minimization [5].

There are a number of potential advantages and disadvantages to the use of minimization for the allocation of participants [6]. In small studies minimization can increase study credibility and value by providing well matched allocation groups. In addition, planning for minimization also allows more participant factors to be taken into account than would be possible with stratified randomization in a small study. A major potential disadvantage is the complexity of the allocation process which is not straightforward without the use of real time centralised computation to analyze the characteristics of the proposed participant and the previously allocated participants. Unlike simple randomization which can be undertaken using sealed envelopes that have a pre-randomized allocation outcome concealed within them, it is not possible to undertake minimization in advance. The requirement for a constantly updated and centralized computer system can be onerous and often prohibitive for a small study run by a small research group or individual with limited research funds. This can be a particular problem in economically disadvantaged countries or studies that have multiple recruitment sites.

The logistical problems associated with the allocation process are likely to be significant factors in the relatively low rate of adoption of minimization. A 2001 review of 150 reports of randomized controlled trials in major journals showed that only 4% used minimization [6]. A detailed 2009 review of articles reporting randomized clinical trials found that only 1.6% of these used minimization, but that the proportion had tripled since the previous decade [7]. Over 500 studies had used minimization in the 10 years preceding the study. It remains a major concern that small studies may lack value because they cannot recruit sufficient numbers to achieve equality of participant characteristics between their control and experimental arms.

In setting up our own small low budget clinical trial we considered that minimization would be a good choice for participant allocation. However, no simple open source or free software was available that was within our budget and that would be easy to implement without complex infrastructure or technical support. In particular, we needed to undertake allocation concurrently over many different sites of different types and these sites did not share any common computer network. This necessitated some form of centralised internet-accessible software that could be used on a range of devices including mobile phones, tablets or desktop computers.

There were some programs available but they did not prove suitable and generally were not freely available in a usable form. An MS-DOS program known as Minim has been used, but can only be run on one computer and does not have any facility for simple remote access, so is not suited to studies recruiting on multiple sites [8]. Furthermore, it does not run on the standard Windows operating systems from Windows 7 onwards and is, therefore, relatively obsolete in its current form. Minim is not open source or free software as, although the compiled executable file is available, the original programming language source code from which it was generated has not been published. A further programme that is only operational on a single computer has been reported and was developed using the now obsolete TurboPascal [9]. A different system developed with a CGI (common gateway interface) has not been made available, is not open source and is no longer testable at the URL (uniform resource locator) in the manuscript [10]. A complex approach has been developed using a relational database operated on a server, but it requires considerable computational infrastructure and expertise to set up and administer, which would add substantially to the cost of its implementation [11]. Furthermore, it is not freely available on an open source or other basis. A further database implementation has been reported as MagMin and is proprietary software that requires the use of an SQL (structured query language) server database and so is also not suitable for small studies with no major computational support [12]. The code for MagMin is not available for scrutiny and it is not open source or free software. MinimPy is written using the Python programming language [13], but requires various libraries and is not suitable for multi-site studies [14].

We were unable to identify any suitable open source software or free software that could be used for study participant allocation by minimization in real time over multiple centres. The case for open source clinical trial data management software has been made [15], [16] and is very strong. It is essential that with any trial allocation procedure the allocation procedure is transparent and CONSORT guidance is that “Authors should provide sufficient information that the reader can assess the methods used to generate the random allocation sequence and the likelihood of bias in group assignment” [5]. If the allocation procedure is based upon computer code that is kept secret from the reader and the investigators then these conditions are not fulfilled. The need for transparency about the computer code used in all scientific studies has been well made and any code that has played a role in the execution of a study or the analysis of the results should ideally be available for scrutiny during peer review and post publication [17]. To address this need and to produce software that could be used for our own studies OxMaR (Oxford Minimization and Randomization) was developed. OxMaR allows randomization or minimization to be done through a simple web page interface from any location via a computer, mobile phone or any other device that can connect to the internet. It is freely available for global use.

## Methods

To maintain portability and ease of implementation, OxMaR was written using standard Perl (v5.10.1) without any additional libraries [18]. Two Perl CGI scripts constitute the complete package and 3 simple text files are used by the scripts. The web interface consists of a single simple HTML web page which can be hosted on any standard web server and viewed with any standard browser on any standard computer, tablet, mobile phone or other device.

The source code is freely available for download from www.ccmp.ox.ac.uk/oxmar or from https://sourceforge.net/projects/oxmar/.

## Results

### Operation for participant allocation

**Figure 1:**
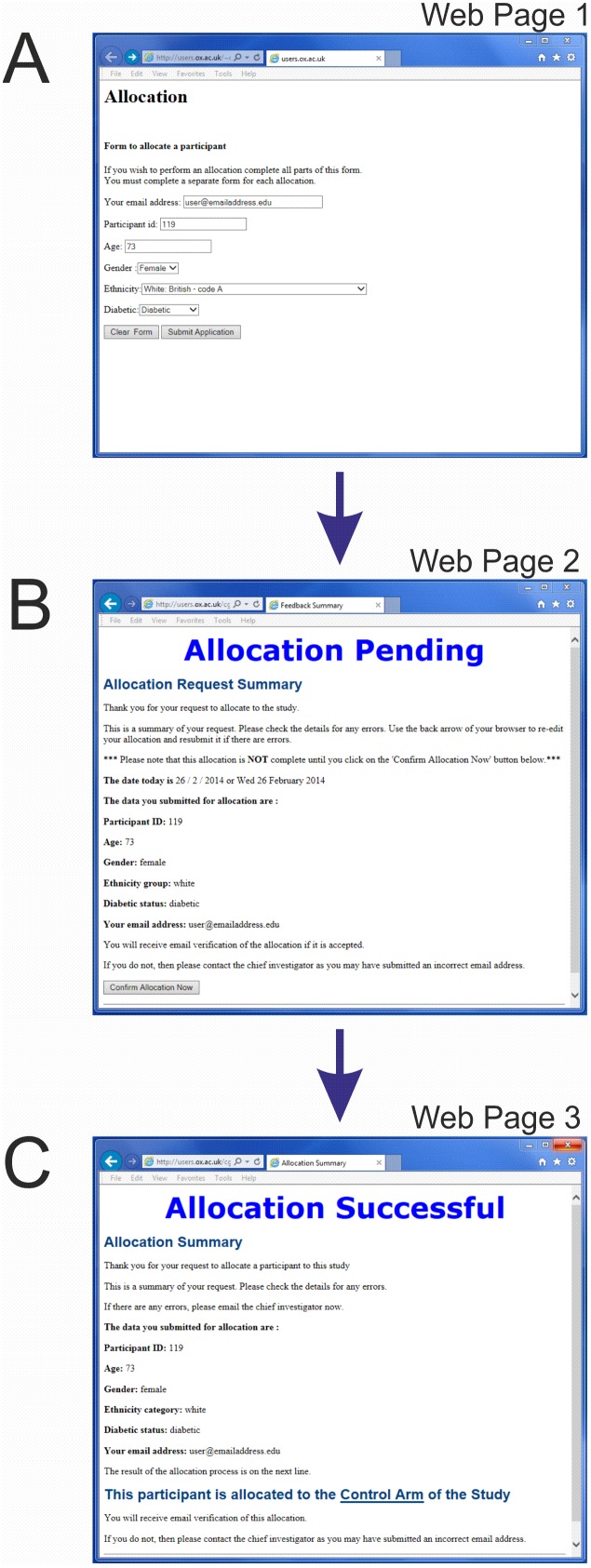
Web-based user interface. Screen images are shown for each of the three web pages that the user will see. The first web page (A) contains a simple form that the user completes and submits. This triggers production of a second web page (B) which contains a summary of the data that has been inputted. The user clicks a button to confirm that this is correct. The allocation procedure is then implemented and a third web page (C) is generated which contains the allocation result. In the example shown, this is to the Control arm of the study.

**Figure 2:**
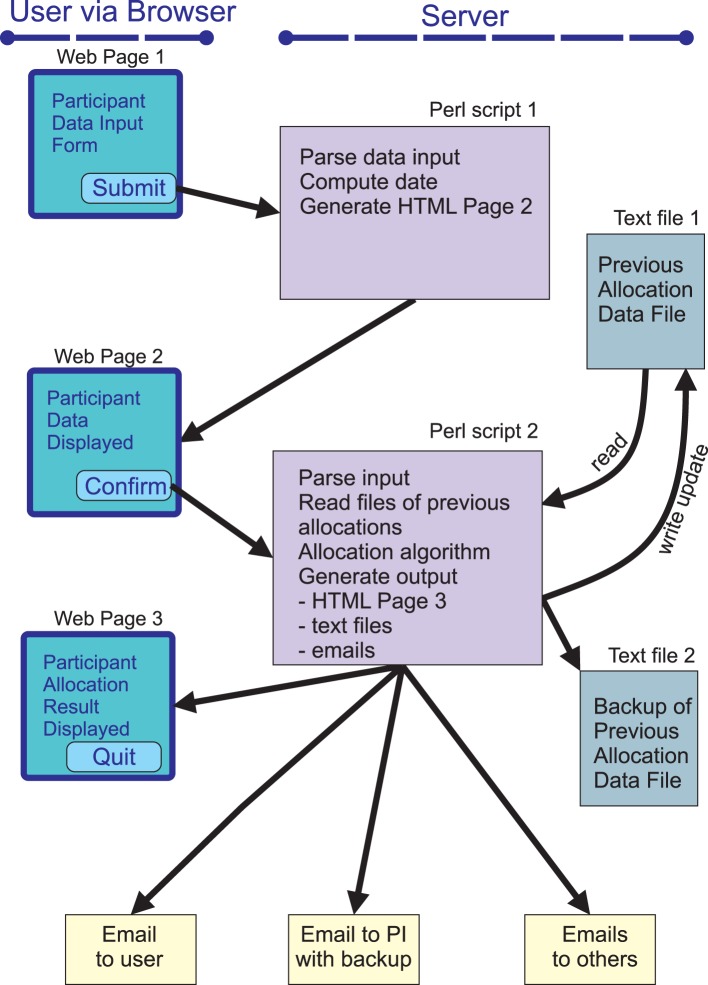
Overview of OxMaR. The system is accessed by the user through a simple web page (Web Page 1). When the form on this page is submitted by the user the first Perl script generates a second web page (Web Page 2) which displays a summary of the data that the user has input. If the user confirms that this is correct, then the information about the new participant is submitted to the second Perl script which performs the allocation procedure. This Perl script reads information from a text file about the previous allocations and generates an allocation decision for the new participant. The script then generates output in the form of a web page (Web Page 3) for the user which informs them of the allocation decision and a series of emails to the user, the principal investigator and one or more other members of the research team. The script also updates the text file with information about the new allocation and writes this to a further backup text file. The email to the principal investigator contains all the information about this and previous participants and so serves as a further backup of the study.

To allocate a participant to a particular group a researcher uses any web browser to navigate to the allocation web page over the internet and completes a simple form (Figure 1A). The web page can be password protected if required. This first web page contains a simple form written in standard HyperText Markup Language (HTML) and the elements of this form can be edited easily according to the requirements of the specific study. The researcher enters data onto the form such as age, gender and participant identification number. The form is then submitted using the common gateway interface (CGI) post method and calls a CGI script written in Perl (Figure 2). This script parses the information in the form, computes the date and weekday and generates a second HTML page which is displayed by the browser (Figure 1B). This second HTML web page summarises the data input from the form and the date and weekday. If the user is happy that the displayed information is correct, then a submit button can be clicked. This then submits the information that was input onto the original form using the CGI post method to call a second CGI script written in Perl (Figure 2). This second CGI script performs the allocation and is the workhorse of the software. The end result is a new HTML web page (Figure 1C) which documents both the submitted information and the study group to which the participant has been allocated. In addition, emails containing the submitted information and the group to which the participant has been allocated are sent to the person submitting the participant and to one or more researchers such as the principal investigator (PI) for their information. By default the email to the principal investigator contains information about all previous allocations and so serves as an automatic real time backup of the entire study (File S1). The results of the allocation are also written to a working text file and a further back up text file. The working text file is read by the second Perl script prior to each allocation to provide the algorithm with information about previous allocations.

### Set-up

A key strength of OxMaR is that it can be used from anywhere in the world through any device that can connect to the internet. This requires that it is run from a webserver. Most academic institutions or medical centres will have their own website and it can easily be hosted on webservers used for this purpose. Where this is not possible a simple web service may need to be purchased, but the cost of this is very low and often free of charge. The service selected needs to allow the use of Perl CGI programming, which is now standard. There are many competing services available as an internet search using the terms ‘web hosting Perl’ will demonstrate.

### Information structure and variables

The first HTML web page can be used to do simple processing of the information, if required. The form can be used to distil data and an example is executed whereby the researcher inputs the precise ethnic category from a choice of 17 detailed options, but the categories are then simplified by allocating each of the 17 options to one of 4 more generic values (eg. ‘White: British – code A’, ‘White: Irish – code B’ and ‘White: Any other White background – code C’ are all aggregated to the category ‘white’). Thus, the form allows the detailed information to be translated into simpler aggregated categories if required.

When the form is submitted, the first CGI Perl script is called and then parses the input data and allocates them to a series of variables (Figure 2). These variables represent the information submitted on the form on the first HTML web page and the computed date and weekday. If the user confirms that the information is correct, the second CGI Perl script is called and the variables are transferred to this script.

Some additional experiment-specific variables are also established. Those implemented include a variable ($agethreshold) which sets an age threshold to divide patients according to age with a default age-dividing setting of 65. A variable $randomisationelement is set to define the extent of the randomization that is to be employed. If this is set to 1 there is no randomization. If it is set to 0 there is totally random allocation without minimization. If it is set to 0.8 there is randomization at the 20% level. Various variables are set to hold email addresses for the administrator and others who should receive information by email about successful allocations. Variables are set to hold file names for the text files that will contain the record of allocations and with it the information about study participants who have been allocated.

**Figure 3:**
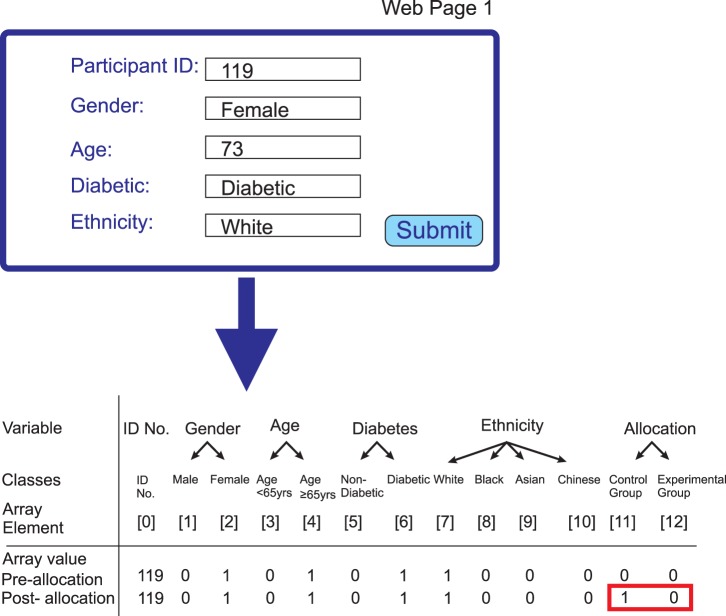
Array used to contain new participant information. The upper panel shows the information in the form that is inputted by the user through the first web page (Web Page 1). When this information is confirmed the second Perl script then parses this information and constructs an array of the form shown. Several variables, such as the participant ID number and date, are included in the array, but are not all shown as they do not play a role in the allocation procedure and serve a purely documentary role. For each variable that is factored into the allocation algorithm, an array element is created for each of the possible options for this variable. Therefore, there are two array elements for gender, one for male and one for female. For a female participant, the value of the element for male will be 0 and the value of the element for female will be 1 as shown. It should be noted that for each variable such as age, gender or ethnicity only one of the value options can be 1 and all others will be 0. The array also contains two elements that will encode the group to which the participant has been allocated. When the array is first created both elements are set to 0, but if, as shown here, the participant is allocated to the control group, then the value of the control group element will then be changed to 1.

The experimental details for the study participants are then set up as an array (Figure 3). The variables that are to be used for minimization are within this array and constitute a series of ‘classes’, each represented by an element of the array. If, for example, there are only two possibilities to be considered, such as male and female, then there are two ‘classes’. Each class will be given a value of 1 if it is true or 0 if it is false. Thus a male would have a value of 1 for the class ‘male’ and a value of 0 for the class ‘female’. If there are a further two possibilities, such as ‘old’ and ‘young’, then there are four ‘classes’ and so on. A total of 10 classes are implemented in the current script and this is easily modified. A variable $numberofclasses is the total number of classes that describes the information used in the allocation procedure. For a standard binary allocation process with an experimental and control arm, two further classes are established that contain the outcome of the allocation process. One class is for ‘option 1’ (the control group) and the other for ‘option 2’ (the experimental group). For each outcome a text string such as ‘Experimental Arm’ is defined which can be used to communicate the outcome to the user and each text string is linked to the appropriate option using a simple hash (% choicehash). A participant allocated to the experimental arm will have a value of 1 for the class corresponding to that arm and a value of 0 for the class corresponding to the control arm, whereas a participant allocated to the control arm will have a value 0 for the class corresponding to experimental arm and a value of 1 for the class corresponding to the control arm.

### Algorithmic execution

The main program runs a series of subroutines with clearly delineated functions. These are called in the following order by the second Perl script to execute the allocation process.

#### Processing of previous allocations

**Figure 4:**
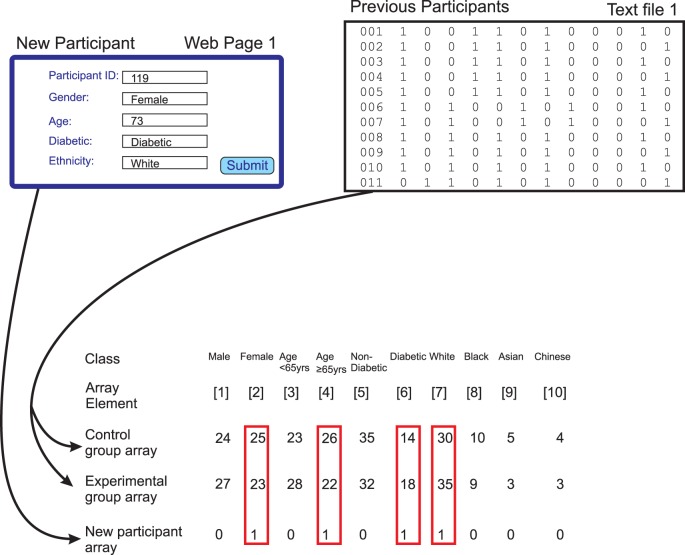
Use of arrays in the allocation process. Information about the new participant is contained in an array of the type illustrated in Figure 3. Information about participants who have previously been allocated is first extracted from a text file. This information is then aggregated into two arrays that have a similar structure to that used for the new participant. One of these arrays aggregates information about participants who have been allocated to the control arm of the study and the other array aggregates information about participants who have been allocated to the experimental arm of the study. The elements from these arrays that correspond to the true values for the new participant (boxed in red) are then extracted for each of the control and experimental arrays. Comparison of the values of these elements in the control and experimental group arrays is used to determine the preferred allocation for the new participant through minimization of the difference between the two groups. In this case the sum of the values for the control group is 95 (25+26+14+30) and the sum of the values for the experimental group is 98 (23+22+18+35) so the new participant will be allocated to the control group.

The *ConvertFileLineToArray* subroutine reads the text file that contains previous allocations, if any, and converts this file into two separate arrays, one for the control group and one for the experimental group (Figure 4). These two arrays have the same structure and each contains the number of elements required to accommodate the different features of the study participants as defined by the classes described above. In the current implementation they have the form illustrated in Figure 4.

The value for each element in each of the two arrays is initially set to zero. Each line of the text file corresponds to one previous participant. As each line is read, the contents of the line are parsed according to the delimiter or divider which by default is arbitrarily set as ‘zz’ and the contents of the line are converted to the elements of the array @formatext. If the contents of the line and so of @formatext indicate that the participant was allocated to the control group, then the value of the class elements for this participant are added to the control group array. For example, if the participant is male, the value of the class element for ‘male’ in the control group array will be incremented by 1. If the contents of the line indicate that the participant in question was allocated to the experimental group, then the appropriate increments are made to the value of the elements of the experimental group array. As each line of the text file is read and its contents allocated to the control or experimental array, the appropriate counter variable is incremented according to whether the line relates to someone in the experimental or control group. When all the previous allocations have been processed, the two arrays contain a summary of the information required to compute the allocation of the new participant whose information has been submitted via the web page (Figure 4).

#### Processing of new participant data

In order to determine the appropriate allocation, the information that has been submitted by the user through the web page form is converted into an array with an analogous structure to that created for the two arms of the study by the *ConvertFileLineToArray* subroutine (Figures 3 and 4). Conversion of the new participant data is executed by the *MakeArrayFromInputData* subroutine. First, an empty local array is created and the value of each array element is set to zero. Each piece of information is then coded into the array. For information such as gender with a binary value the array element for ‘GenderMale’ is given a value 1 and the array element for ‘GenderFemale’ is given an array value 0 for a male participant. For a female participant the array element for ‘GenderMale’ is given a value 0 and the array element for ‘GenderFemale’ is given an array value 1. Similarly, for information such as ethnicity with multiple possible values where all ethnicity elements are given the value 0 except for the correct ethnicity which is given the value 1. Overall, all elements that will be used for minimization are given a value of 0 or 1 corresponding to ‘true’ or ‘false’ for this element; these elements are termed allocation elements. Elements such as participant ID are given their real values. The two elements corresponding to the possible outcomes, outcome 1 and outcome 2, of the allocation process are both given the value zero at this stage. The resulting array then contains the information from the form in a format that can readily be used together with the two arrays representing the control and experimental arms of the study to compute the allocation outcome.

#### The Minimization process

The minimization algorithm is executed by the *DoMinimisationAndAllocation* subroutine using the two arrays that have been created from the textfile of previous allocations and the array that has been created from information submitted via the web page about the new participant (Figure 4).

For all array elements for the new participant which have a value of 1, the values of the equivalent array elements in each of the control and experimental group arrays are summated separately (Figure 4). The result is a score for each of the two arrays that represents the frequency of the features of the new participant in each of the control and experimental groups. That is for all allocation elements that have a value of 1 (indicating ‘true’) for the new study participant under consideration, then the values of those element are added up for each of the control and experimental group independently.

The scores for each study group (control and experimental group) are then compared. Three scenarios are then possible. Firstly, the score for the control group and the score for the experimental group may be equal, in which case the new participant is allocated to one of the two groups randomly. This will always be the case for the first participant to be allocated who will therefore always be randomized. Secondly, the score for the experimental group may be greater than that for the control group, in which case the new participant is allocated to the control group. Thirdly, the score for the control group may be greater than that for the experimental group, in which case the new participant is allocated to the experimental group. In the latter two cases, the allocation may be subjected to a degree of randomization to the extent specified for the study by the variable $randomisationelement as discussed above. Overall, the effect of the algorithm is to minimize differences between the two groups in the distribution of the characteristics of the new participant being allocated.

#### Randomization in the allocation process

In all three cases, the actual allocation is achieved by a call to the *RandomiseThisParticipant* subroutine. For complete randomization this call is given with the parameter 0.5. For allocation to one group the parameter is set at 1 and for allocation to the other group the parameter is set at 0. By default, the variable $randomisationelement is set to 1. Therefore, for allocation to option 1, the control group, the call is given with the parameter $randomisationelement. For allocation to option 2, the experimental group, the call is given with the parameter 1-$randomisationelement. The output of the *RandomiseThisParticipant* subroutine is a selection of ‘option 1’ or ‘option 2’ with an algorithm that incorporates the degree of randomization dictated by the variable $randomisationelement. This variable formally defines the probability that the outcome will be option 1 when it is used as the input parameter for the $RandomiseThisParticipant subroutine and so defines the threshold that is used to determine allocation on the basis of a randomly generated number. If the randomly generated number is below the threshold determined by the input parameter, then option 1 is selected. Otherwise, if it is greater than this, option 2 is selected. Therefore, with an input parameter of 1, the probability of option 1 is 100%, with an input parameter of 0, the probability of option 1 is 0 and so the probability of option 2 is 100%. With an input parameter of 0.5 a random choice is made between option 1 and option 2. If a completely random assignment is required, then $randomisationelement can be set to 0.5. Overall, the *DoMinimisationAndAllocation* subroutine calls the *RandomiseThisParticipant* subroutine and outputs an allocation choice for the participant as ‘option 1’ or ‘option 2’.

As well as the option to use complete randomization, other options are also easily implemented. For example, if the different allocation classes are to be given different weights, this can be simply achieved by altering the numerical value allocated for a true value in that element of the array. If ethnicity was to be only half as important as gender, then the numerical value for ethnicity could be altered to 0.5 instead of 1.

### Generating output

Following selection of the appropriate choice, the array that contains information about the new participant is updated by the *ReviseParticipantArrayWithResult* subroutine with the allocation choice for this participant. As illustrated in Figure 4, the array element corresponding to the chosen arm of the study is given the value 1. The *MakeArrayOneLine* subroutine then converts this array into a suitable format for writing to a file by generating a line of text in which the individual elements are delineated by the delimiter or divider which is arbitrarily set as ‘zz’. The *FileWrite* subroutine then appends the line that contains the information about the new participant to the text file that contains information about the previous participants (Figure 2). A duplicate set of information is also appended to an archive file that is not routinely read. The main program then interrogates the hash $choicehash to establish the text (eg. ‘Control Arm’) to use to describe the allocation in subsequent outputs including the final HTML web page.

A simple HTML web page (Figure 1C) is constructed and displayed by the *MakeHTMLPageSuccess* subroutine. This web page summarises the details submitted by the user about the new participant and the outcome of the allocation process - whether the participant will be in the control or experimental group. Simple subroutines are called to produce headers and footers for the HTML web pages, which facilitates the creation of a uniform appearance between the different HTML web pages produced during the allocation process. The *WhatTimeNow* subroutine computes the current time.

The emails to be sent are then composed by the *SendEmails* subroutine. By default three emails are sent (Figure 2). The first email is sent to the email address that the researcher submitted though the web page form. This email summarises the information submitted via the form, reports the time of the allocation and the outcome of the allocations. It also enumerates the total number of participants that have been allocated and the number allocated to the control arm and the experimental arm. The second email is sent by default to the study nurse and is essentially a copy of the first email. The third email is sent to the principal investigator or study administrator. This email includes the information that is in the previous emails, but in addition it contains the contents of the line of text added to the text file and all the contents of the text file. Thus, it serves as an entirely independent backup of the entire study allocation history and so would allow the study to be reconstructed in the absence of the text files if necessary. The main program then sends these emails to the different recipients with a subject line that includes the date. This concludes the allocation process with outputs to a web page that the user can see, to text files on the server and to a series of email addresses.

### Field testing and validation

The software has been extensively tested using a range of devices including standard desktop PCs, Apple Mac computers, tablets and mobile phones and has worked well with all devices. It has now been used in the OxCKD1 study which is ongoing and has now recruited over 150 patients using minimization with OxMaR to allocate them to a control or experimental arm of the study. These allocations have been conducted from a variety of locations including hospitals, university and community-based locations. The software has proved robust and after initial testing there have been no bugs or crashes identified during 18 months of use in this clinical trial. The value of the minimization approach can be seen in the distribution of patient characteristics between the control and experiment group (Table 1).

**Table 1:** Characteristics of allocated participants in OxCKD1.

		ControlGroup	Experimental Group
**Gender**	Male	38	38
	Female	38	38
**Age**	<65 years	36	35
	≥65 years	40	41
**Diabetes**	No	56	57
	Yes	20	19
**Ethnicity**	White	72	70
	Black	2	3
	Asian	1	3
	Chinese	1	0

## Discussion

The value of simple randomization as a method for allocating participants between the control and experimental arms of a study is well recognised. However, it is also well recognised that under certain circumstances simple randomization may not perfectly distribute multiple participant characteristics such as age, gender or ethnicity equally between the arms of the study. This problem arises principally in small studies and can give rise to unreliable study outcomes that may reflect the distribution of participant characteristics rather than the effect or otherwise of an intervention. The occurrence of this confounding problem has not been quantified, but it is likely to be a major reason why the results of small, ostensibly similar, trials can differ in their outcome.

The method of allocation by minimization has been developed to address this problem [1], [2]. A major conceptual difference between randomization and minimization is that in minimization, the characteristics of participants who have already been allocated to each study arm are taken into consideration in the allocation process, using an algorithm that attempts to minimize differences between the two study arms. In randomization each allocation is determined independently and randomly without consideration of previous allocations.

This conceptual difference has major practical and logistical consequences which have made randomization superficially attractive, even for studies for which it may not be the best allocation approach. For randomization, study arm allocation can proceed in real time without the need for any significant study infrastructure or support. Indeed, in its most simple form, randomization can be performed by opening a sealed envelope to find out which study arm the participant has been allocated to. Multiple centres can recruit simultaneously by randomization without any real time co-ordination and so can function autonomously once the study is established.

In marked contrast, minimization requires significant real time computation for the allocation of each participant. In addition, multiple sites need to able to access real time information about all previous allocations, including those at other sites, in order to perform an allocation. Historically, these limitations have likely restricted the use of minimization, when it would otherwise have been a preferred choice for study arm allocation. The problem is accentuated because the very studies that would most benefit from the use of minimization are smaller studies, which are most likely to lack complex infrastructural or computational support, especially in resource-poor environments.

Nevertheless, rapid developments in technology have led to the availability of progressively cheaper access to the internet through mobile phones and other devices, even in poorer regions of the world. In parallel, access to web hosting services has become extremely cheap and often free, both within academic and other research institutions and beyond, with many commercial companies now offering free access to web servers.

These developments should allow the more widespread use of minimization. Unfortunately, there is currently no freely available simple open source or free software that can be used to run a study using minimization for participant allocation across multiple sites. Given the computational nature of the minimization allocation process, it is essential that any software code used to allocate patients is available as open source code for scrutiny so that the allocation procedure is explicit [5]. Therefore OxMaR was developed as an open source minimization software package and tested and validated in our own study. This demonstrates that it works very well at distributing participant characteristics equitably between study arms, thus potentially adding substantial value to studies where this might not happen by chance.

In developing OxMar a key objective was to produce software that was transparently open to scrutiny and peer review and that was available as a resource for other workers in unrestricted form as free software. The source code is available as open source and free software from www.ccmp.ox.ac.uk/oxmar or from https://sourceforge.net/projects/oxmar/.

The OxMaR system has a number of strengths. It is robust and uses highly standard and relatively future proof code throughout. Once set up, it can be run without any need to deal with the server other than through the simple web pages of the software. This arrangement would therefore allow it to be set up by a collaborator or study group member who is distant from the study and who does not need to be involved subsequently. OxMaR can be used from any device that can access the internet, such as a desktop, laptop, tablet or phone. The pages that it delivers are very simple and so load quickly and clearly even with relatively slow connections. A key strength is that with each allocation, emails are sent out from which it is possible to reconstruct the study with ease, even if all the files on the server were to be lost. Thus there is effectively a distributed real time back up within the cloud. This means that the researchers have access to all their data at all times, even if the server were to crash. The centralised nature of OxMaR means that it is ideal for multicenter studies, even if those sites are distributed around the globe. Although the allocation procedure factors in the previous allocations, information about these allocations can easily be concealed from the person undertaking the allocation and is done so by default. Indeed the nature of the computation involved in minimization makes it unlikely that a researcher could easily predict the likely allocation of a patient by the algorithm and in multicenter studies they would not know whether someone else had been allocated since their own last allocation.

A range of customisations are possible and the open nature of the software lends itself to these options. At a basic level, it is possible to alter the number of participant characteristics that are factored into the allocation algorithm. In addition, different weightings can be given to different characteristics by altering the value of the score given for each characteristic. The files produced or the information in the emails sent with each allocation can be imported easily into a spreadsheet or other software packages for further analysis. The extent of randomization required within the minimization algorithm can be customised easily. Furthermore, if complete randomization is required and the study is to be a standard randomized controlled trial then this can be trivially implemented by adjusting the randomization element to the value required for complete randomization. This could be a useful option for studies that have multiple centres as it would provide central information on study allocation progress. The real time processing of allocation in this case, as with minimization, provides a central record of the allocation, which is documented in real time and which cannot be tampered with locally.

In some situations, internet connections may be slow or relatively costly and OxMaR is designed to maximize utility and minimize costs in this context. The software produces very simple web pages encoded by brief basic HTML and the content of the web forms is similarly simple so that data transmission requirements are very low. Any limited service will be sufficient to undertake the study and in extreme situations, a field worker could phone or text data to the study base or other location, supplying the information required for someone at that location to input the participant variables into the web page to generate an allocation. In the situation where there are no phone or internet services at all, it is not possible to undertake a multi-centre study using real time minimization as there is an absolute requirement for real time information about the previous study participants in order to perform the minimization process. Under these circumstances, there are two options. One is to undertake a single centre study using local computation and this could easily be done using OxMar installed directly on a single desktop or portable computer. The second is to acquire participant information locally and then take this to an area where there is a suitable phone or internet connection and then return subsequently with the results of the allocation procedure. However, mobile phone coverage is increasing rapidly, even in poor countries, so this issue will be a diminishing one. In areas where it is especially problematic, the use of satellite phones is increasingly satisfactory and the costs are not substantial compared to those with standard mobile phone services.

The email sent out to the person submitting the following an allocation is a simple text email containing 18 lines of text that states the variables submitted and the outcome result. It does not contain a summary of the data relating to previous allocations. As such this email requires only minimal data transmission and it is very simple to reduce this email further. In addition, it is not necessary for the person submitting the allocation request to review the email immediately and this email might simply be viewed for confirmation at a later stage, perhaps on return to the study base. To reduce data transmission requirements phones or remote device preferences could be set to only downloaded emails at preferred times or on request, such as only when free or low cost connections are available.

By contrast the email to the study administrator and potentially to other individuals does contain a copy of the entire dataset. This should not be an issue for data transmission as these emails will only need to be looked at when the administrator chooses to do so and the email does not play any specific role in the routine running of the allocations. Nevertheless, the information in this email is in the form of simple compact text and should not be problematic from a data transmission perspective. If this does constitute a problem, the simplest solution would be for it to be sent to a free web-based email account that is only looked at intermittently through a browser at a location where data transmission is not a problem.

The OxMaR algorithm is coded in the standard implemented programming language Perl [18], which does not require compilation and so can be read intuitively and so scrutinised straightforwardly. No external packages or libraries are used so every detail of the implementation is available for inspection in line with CONSORT principles and those outlined by the International Conference on Harmonisation [5], [19]. This also makes further customization straightforward as all aspects of the software are available to the user directly.

Setting up a study with OxMaR is relatively straightforward and, if necessary, can be done at zero cost using free web hosting services. Only minimal, if any, adjustment of the files is required and clear instructions are provided about this. Furthermore, the software is available on an open source basis as free software which allows others beyond the author to develop the software further, customise it and support users in their deployments. OxMaR is publicly available to be built on and customised – the basic engine has been demonstrated to be robust and can be deployed globally. This platform should be of immediate utility but should also allow development of the software to meet the needs of future users with specific requirements. OxMaR is a useful solution even for very low budget studies and should help researchers to retain their resources to spend on other aspects of the study rather than software support. It may also be of value in studies undertaken by students, nurses and other health professionals, who may not always have access to the levels of funding available for standard or commercial drug trials. In particular, it is hoped that the software will be of value to resource-poor research communities, whether they be in in any field in poorer nations or in fields not favoured by funding bodies in prosperous nations.

## Supporting Information

File S1 **Supplementary information file containing examples of the emails that are sent out by OxMaR after each allocation, an example of the text file written by OxMaR and typical HTML code for the first web page.**
(DOCX)
