# Supplementary material for: OxMaR: Open Source Free Software for Online Minimization and Randomization for Clinical Trials
Source: PLoS One. 2014 Oct 29;9(10):e110761. doi: 10.1371/journal.pone.0110761 (PMC4213009; doi:10.1371/journal.pone.0110761)
Supplement: File S1 — Supplementary information file containing examples of the emails that are sent out by OxMaR after each allocation, an example of the text file written by OxMaR and typical HTML code for the first web page. (DOCX) [file pone.0110761.s001.docx]

**Supplementary information**

Email to study nurse

Dear Study Nurse,

This email is to inform you that a study allocation request was made on Wed 26/2/2014 at 18:15.

Note - this is GMT - add one hour if the hour has gone forward for British Summer Time (BST) If the time in this email is 20:55 that will be 21:55 during BST.

The submitted data are below:

Participant ID: 119

Age: 73

Gender: female

Ethnicity category: white

Diabetic status: diabetic

The request was accepted and this participant has been allocated to the Control Arm of the Study.

If there is a problem or error please email [chris.ocallaghan@ndm.ox.ac.uk](mailto:chris.ocallaghan@ndm.ox.ac.uk) immediately. Do not reply to this email as the email address will not reach anyone.

For your information, this participant is the 47 th/st/rd participant to be allocated.

The total number of participants previously allocated to the control arm is 23.

The total number of participants previously allocated to the interventional arm is 23.

Best wishes,

The Allocation Script

Email to study administrator or principal investigator

Dear Administrator,

An allocation was requested on

Wed 26/2/2014 at 18:15.

Note - this time is GMT - add one hour if the hour has gone forward for British Summer Time (BST) ie if the time in this email is 20:55 that will be 21:55 during BST

The submitted data are below:

Participant ID: 119

Age: 73

Gender: female

Ethnicity category: white

Diabetic status: diabetic

This participant has been allocated to the Control Arm of the Study.

The data line for this participant is below.

119 zz 0 zz 1 zz 0 zz 1 zz 0 zz 1 zz 1 zz 0 zz 0 zz 0 zz 1 zz 0 zz 26 zz 2 zz 2014 zz 73 zz

This participant is the 47 th/st/rd participant to be allocated.

The total number of participants previously allocated to the control arm is 23.

The total number of participants previously allocated to the interventional arm is 23.

The preceding dataset is

001 zz 0 zz 1 zz 1 zz 0 zz 1 zz 0 zz 1 zz 0 zz 0 zz 0 zz 1 zz 0 zz 6 zz 9 zz 2012 zz 62 zz

003 zz 1 zz 0 zz 1 zz 0 zz 0 zz 1 zz 1 zz 0 zz 0 zz 0 zz 0 zz 1 zz 10 zz 9 zz 2012 zz 59 zz

002 zz 1 zz 0 zz 1 zz 0 zz 1 zz 0 zz 1 zz 0 zz 0 zz 0 zz 1 zz 0 zz 17 zz 9 zz 2012 zz 53 zz

004 zz 0 zz 1 zz 1 zz 0 zz 0 zz 1 zz 1 zz 0 zz 0 zz 0 zz 0 zz 1 zz 24 zz 10 zz 2012 zz 34 zz

005 zz 1 zz 0 zz 1 zz 0 zz 1 zz 0 zz 1 zz 0 zz 0 zz 0 zz 0 zz 1 zz 25 zz 10 zz 2012 zz 64 zz

007 zz 1 zz 0 zz 0 zz 1 zz 1 zz 0 zz 1 zz 0 zz 0 zz 0 zz 1 zz 0 zz 28 zz 11 zz 2012 zz 74 zz

008 zz 0 zz 1 zz 0 zz 1 zz 1 zz 0 zz 1 zz 0 zz 0 zz 0 zz 0 zz 1 zz 28 zz 11 zz 2012 zz 72 zz

008 zz 0 zz 1 zz 0 zz 1 zz 1 zz 0 zz 1 zz 0 zz 0 zz 0 zz 1 zz 0 zz 28 zz 11 zz 2012 zz 72 zz

009 zz 0 zz 1 zz 1 zz 0 zz 1 zz 0 zz 1 zz 0 zz 0 zz 0 zz 0 zz 1 zz 5 zz 12 zz 2012 zz 64 zz

009 zz 0 zz 1 zz 1 zz 0 zz 1 zz 0 zz 1 zz 0 zz 0 zz 0 zz 1 zz 0 zz 5 zz 12 zz 2012 zz 64 zz

006 zz 1 zz 0 zz 1 zz 0 zz 1 zz 0 zz 1 zz 0 zz 0 zz 0 zz 0 zz 1 zz 5 zz 12 zz 2012 zz 64 zz

010 zz 0 zz 1 zz 1 zz 0 zz 0 zz 1 zz 1 zz 0 zz 0 zz 0 zz 1 zz 0 zz 8 zz 1 zz 2013 zz 50 zz

012 zz 1 zz 0 zz 0 zz 1 zz 0 zz 1 zz 1 zz 0 zz 0 zz 0 zz 1 zz 0 zz 8 zz 1 zz 2013 zz 70 zz

017 zz 1 zz 0 zz 0 zz 1 zz 1 zz 0 zz 1 zz 0 zz 0 zz 0 zz 0 zz 1 zz 8 zz 1 zz 2013 zz 79 zz

019 zz 0 zz 1 zz 0 zz 1 zz 0 zz 1 zz 1 zz 0 zz 0 zz 0 zz 0 zz 1 zz 8 zz 1 zz 2013 zz 73 zz

015 zz 0 zz 1 zz 1 zz 0 zz 1 zz 0 zz 0 zz 0 zz 0 zz 1 zz 1 zz 0 zz 9 zz 1 zz 2013 zz 45 zz

016 zz 1 zz 0 zz 0 zz 1 zz 1 zz 0 zz 1 zz 0 zz 0 zz 0 zz 1 zz 0 zz 9 zz 1 zz 2013 zz 67 zz

012 zz 0 zz 1 zz 0 zz 1 zz 1 zz 0 zz 1 zz 0 zz 0 zz 0 zz 0 zz 1 zz 9 zz 1 zz 2013 zz 70 zz

013 zz 0 zz 1 zz 1 zz 0 zz 1 zz 0 zz 0 zz 1 zz 0 zz 0 zz 0 zz 1 zz 9 zz 1 zz 2013 zz 53 zz

014 zz 0 zz 1 zz 0 zz 1 zz 1 zz 0 zz 1 zz 0 zz 0 zz 0 zz 1 zz 0 zz 9 zz 1 zz 2013 zz 73 zz

020 zz 0 zz 1 zz 1 zz 0 zz 1 zz 0 zz 1 zz 0 zz 0 zz 0 zz 1 zz 0 zz 14 zz 1 zz 2013 zz 62 zz

018 zz 1 zz 0 zz 0 zz 1 zz 1 zz 0 zz 1 zz 0 zz 0 zz 0 zz 0 zz 1 zz 14 zz 1 zz 2013 zz 68 zz

021 zz 1 zz 0 zz 0 zz 1 zz 1 zz 0 zz 1 zz 0 zz 0 zz 0 zz 1 zz 0 zz 14 zz 1 zz 2013 zz 76 zz

022 zz 1 zz 0 zz 0 zz 1 zz 1 zz 0 zz 0 zz 0 zz 1 zz 0 zz 0 zz 1 zz 14 zz 1 zz 2013 zz 82 zz

023 zz 1 zz 0 zz 0 zz 1 zz 1 zz 0 zz 1 zz 0 zz 0 zz 0 zz 0 zz 1 zz 16 zz 1 zz 2013 zz 71 zz

024 zz 0 zz 1 zz 0 zz 1 zz 1 zz 0 zz 1 zz 0 zz 0 zz 0 zz 1 zz 0 zz 16 zz 1 zz 2013 zz 68 zz

025 zz 1 zz 0 zz 1 zz 0 zz 1 zz 0 zz 1 zz 0 zz 0 zz 0 zz 1 zz 0 zz 16 zz 1 zz 2013 zz 62 zz

027 zz 0 zz 1 zz 1 zz 0 zz 1 zz 0 zz 1 zz 0 zz 0 zz 0 zz 0 zz 1 zz 22 zz 1 zz 2013 zz 40 zz

028 zz 0 zz 1 zz 1 zz 0 zz 1 zz 0 zz 0 zz 1 zz 0 zz 0 zz 0 zz 1 zz 23 zz 1 zz 2013 zz 45 zz

026 zz 0 zz 1 zz 0 zz 1 zz 1 zz 0 zz 1 zz 0 zz 0 zz 0 zz 0 zz 1 zz 23 zz 1 zz 2013 zz 75 zz

029 zz 1 zz 0 zz 1 zz 0 zz 1 zz 0 zz 1 zz 0 zz 0 zz 0 zz 1 zz 0 zz 30 zz 1 zz 2013 zz 62 zz

031 zz 0 zz 1 zz 1 zz 0 zz 1 zz 0 zz 1 zz 0 zz 0 zz 0 zz 1 zz 0 zz 1 zz 2 zz 2013 zz 46 zz

030 zz 0 zz 1 zz 1 zz 0 zz 1 zz 0 zz 1 zz 0 zz 0 zz 0 zz 0 zz 1 zz 4 zz 2 zz 2013 zz 33 zz

032 zz 0 zz 1 zz 0 zz 1 zz 1 zz 0 zz 1 zz 0 zz 0 zz 0 zz 1 zz 0 zz 5 zz 2 zz 2013 zz 67 zz

034 zz 1 zz 0 zz 1 zz 0 zz 1 zz 0 zz 1 zz 0 zz 0 zz 0 zz 0 zz 1 zz 25 zz 2 zz 2013 zz 48 zz

035 zz 0 zz 1 zz 0 zz 1 zz 0 zz 1 zz 1 zz 0 zz 0 zz 0 zz 1 zz 0 zz 27 zz 2 zz 2013 zz 75 zz

036 zz 1 zz 0 zz 0 zz 1 zz 1 zz 0 zz 1 zz 0 zz 0 zz 0 zz 0 zz 1 zz 27 zz 2 zz 2013 zz 75 zz

040 zz 0 zz 1 zz 0 zz 1 zz 0 zz 1 zz 1 zz 0 zz 0 zz 0 zz 0 zz 1 zz 14 zz 3 zz 2013 zz 77 zz

041 zz 1 zz 0 zz 1 zz 0 zz 0 zz 1 zz 1 zz 0 zz 0 zz 0 zz 1 zz 0 zz 20 zz 3 zz 2013 zz 62 zz

041 zz 1 zz 0 zz 1 zz 0 zz 0 zz 1 zz 1 zz 0 zz 0 zz 0 zz 0 zz 1 zz 20 zz 3 zz 2013 zz 62 zz

039 zz 1 zz 0 zz 0 zz 1 zz 0 zz 1 zz 1 zz 0 zz 0 zz 0 zz 1 zz 0 zz 20 zz 3 zz 2013 zz 69 zz

043 zz 1 zz 0 zz 0 zz 1 zz 1 zz 0 zz 1 zz 0 zz 0 zz 0 zz 1 zz 0 zz 20 zz 3 zz 2013 zz 74 zz

044 zz 1 zz 0 zz 1 zz 0 zz 1 zz 0 zz 1 zz 0 zz 0 zz 0 zz 0 zz 1 zz 26 zz 3 zz 2013 zz 48 zz

042 zz 0 zz 1 zz 1 zz 0 zz 1 zz 0 zz 1 zz 0 zz 0 zz 0 zz 1 zz 0 zz 27 zz 3 zz 2013 zz 60 zz

045 zz 1 zz 0 zz 0 zz 1 zz 0 zz 1 zz 1 zz 0 zz 0 zz 0 zz 0 zz 1 zz 3 zz 4 zz 2013 zz 70 zz

046 zz 1 zz 0 zz 1 zz 0 zz 1 zz 0 zz 1 zz 0 zz 0 zz 0 zz 1 zz 0 zz 8 zz 4 zz 2013 zz 56 zz

048 zz 1 zz 0 zz 1 zz 0 zz 1 zz 0 zz 1 zz 0 zz 0 zz 0 zz 0 zz 1 zz 10 zz 4 zz 2013 zz 63 zz

047 zz 1 zz 0 zz 1 zz 0 zz 1 zz 0 zz 1 zz 0 zz 0 zz 0 zz 1 zz 0 zz 10 zz 4 zz 2013 zz 61 zz

049 zz 1 zz 0 zz 0 zz 1 zz 1 zz 0 zz 1 zz 0 zz 0 zz 0 zz 0 zz 1 zz 16 zz 4 zz 2013 zz 73 zz

051 zz 1 zz 0 zz 0 zz 1 zz 1 zz 0 zz 1 zz 0 zz 0 zz 0 zz 1 zz 0 zz 25 zz 4 zz 2013 zz 83 zz

050 zz 1 zz 0 zz 1 zz 0 zz 1 zz 0 zz 1 zz 0 zz 0 zz 0 zz 0 zz 1 zz 1 zz 5 zz 2013 zz 58 zz

054 zz 0 zz 1 zz 0 zz 1 zz 1 zz 0 zz 1 zz 0 zz 0 zz 0 zz 0 zz 1 zz 8 zz 5 zz 2013 zz 75 zz

055 zz 0 zz 1 zz 1 zz 0 zz 1 zz 0 zz 1 zz 0 zz 0 zz 0 zz 1 zz 0 zz 20 zz 5 zz 2013 zz 49 zz

056 zz 1 zz 0 zz 1 zz 0 zz 1 zz 0 zz 1 zz 0 zz 0 zz 0 zz 1 zz 0 zz 22 zz 5 zz 2013 zz 57 zz

057 zz 1 zz 0 zz 1 zz 0 zz 1 zz 0 zz 1 zz 0 zz 0 zz 0 zz 0 zz 1 zz 3 zz 6 zz 2013 zz 25 zz

059 zz 0 zz 1 zz 1 zz 0 zz 1 zz 0 zz 1 zz 0 zz 0 zz 0 zz 0 zz 1 zz 5 zz 6 zz 2013 zz 41 zz

061 zz 0 zz 1 zz 0 zz 1 zz 1 zz 0 zz 1 zz 0 zz 0 zz 0 zz 1 zz 0 zz 12 zz 6 zz 2013 zz 73 zz

062 zz 1 zz 0 zz 1 zz 0 zz 1 zz 0 zz 1 zz 0 zz 0 zz 0 zz 1 zz 0 zz 18 zz 6 zz 2013 zz 30 zz

063 zz 1 zz 0 zz 1 zz 0 zz 1 zz 0 zz 1 zz 0 zz 0 zz 0 zz 0 zz 1 zz 18 zz 6 zz 2013 zz 57 zz

066 zz 0 zz 1 zz 0 zz 1 zz 0 zz 1 zz 1 zz 0 zz 0 zz 0 zz 0 zz 1 zz 19 zz 6 zz 2013 zz 72 zz

064 zz 1 zz 0 zz 1 zz 0 zz 0 zz 1 zz 1 zz 0 zz 0 zz 0 zz 1 zz 0 zz 24 zz 6 zz 2013 zz 48 zz

065 zz 1 zz 0 zz 0 zz 1 zz 1 zz 0 zz 1 zz 0 zz 0 zz 0 zz 1 zz 0 zz 24 zz 6 zz 2013 zz 75 zz

068 zz 0 zz 1 zz 1 zz 0 zz 1 zz 0 zz 1 zz 0 zz 0 zz 0 zz 0 zz 1 zz 25 zz 6 zz 2013 zz 44 zz

067 zz 0 zz 1 zz 1 zz 0 zz 1 zz 0 zz 1 zz 0 zz 0 zz 0 zz 1 zz 0 zz 27 zz 6 zz 2013 zz 63 zz

070 zz 0 zz 1 zz 1 zz 0 zz 0 zz 1 zz 1 zz 0 zz 0 zz 0 zz 0 zz 1 zz 8 zz 7 zz 2013 zz 26 zz

069 zz 1 zz 0 zz 1 zz 0 zz 1 zz 0 zz 1 zz 0 zz 0 zz 0 zz 0 zz 1 zz 15 zz 7 zz 2013 zz 54 zz

073 zz 0 zz 1 zz 0 zz 1 zz 1 zz 0 zz 1 zz 0 zz 0 zz 0 zz 1 zz 0 zz 15 zz 7 zz 2013 zz 69 zz

074 zz 0 zz 1 zz 0 zz 1 zz 1 zz 0 zz 1 zz 0 zz 0 zz 0 zz 0 zz 1 zz 15 zz 7 zz 2013 zz 67 zz

071 zz 1 zz 0 zz 1 zz 0 zz 1 zz 0 zz 1 zz 0 zz 0 zz 0 zz 1 zz 0 zz 18 zz 7 zz 2013 zz 44 zz

075 zz 1 zz 0 zz 1 zz 0 zz 1 zz 0 zz 1 zz 0 zz 0 zz 0 zz 0 zz 1 zz 22 zz 7 zz 2013 zz 53 zz

076 zz 1 zz 0 zz 0 zz 1 zz 1 zz 0 zz 1 zz 0 zz 0 zz 0 zz 1 zz 0 zz 1 zz 8 zz 2013 zz 67 zz

085 zz 0 zz 1 zz 0 zz 1 zz 1 zz 0 zz 1 zz 0 zz 0 zz 0 zz 0 zz 1 zz 28 zz 8 zz 2013 zz 79 zz

082 zz 0 zz 1 zz 1 zz 0 zz 1 zz 0 zz 1 zz 0 zz 0 zz 0 zz 1 zz 0 zz 28 zz 8 zz 2013 zz 64 zz

079 zz 0 zz 1 zz 1 zz 0 zz 1 zz 0 zz 1 zz 0 zz 0 zz 0 zz 0 zz 1 zz 28 zz 8 zz 2013 zz 46 zz

078 zz 1 zz 0 zz 1 zz 0 zz 1 zz 0 zz 1 zz 0 zz 0 zz 0 zz 1 zz 0 zz 29 zz 8 zz 2013 zz 64 zz

084 zz 1 zz 0 zz 0 zz 1 zz 1 zz 0 zz 0 zz 0 zz 1 zz 0 zz 0 zz 1 zz 2 zz 9 zz 2013 zz 66 zz

086 zz 0 zz 1 zz 1 zz 0 zz 0 zz 1 zz 1 zz 0 zz 0 zz 0 zz 1 zz 0 zz 9 zz 9 zz 2013 zz 41 zz

087 zz 1 zz 0 zz 1 zz 0 zz 1 zz 0 zz 1 zz 0 zz 0 zz 0 zz 0 zz 1 zz 11 zz 9 zz 2013 zz 62 zz

083 zz 1 zz 0 zz 1 zz 0 zz 1 zz 0 zz 1 zz 0 zz 0 zz 0 zz 1 zz 0 zz 16 zz 9 zz 2013 zz 63 zz

Best wishes,

The Allocation Script

Email to person submitting the allocation request

Dear Researcher

Thank you for submitting your study allocation request on Wed 26/2/2014 at 18:15.

Note this is GMT - add one hour if the hour has gone forward for British Summer Time (BST) If the time in this email is 20:55 that will be 21:55 during BST.

The submitted data are below:

Participant ID: 119

Age: 73

Gender: female

Ethnicity category: white

Diabetic status: diabetic

Your request has been accepted and this participant has been allocated to the Control Arm of the Study.

If there is a problem or error please email [chris.ocallaghan@ndm.ox.ac.uk](mailto:chris.ocallaghan@ndm.ox.ac.uk) immediately. Do not reply to this email as the email address will not reach anyone.

This participant is the 47 th/st/rd participant to be allocated.

The total number of participants previously allocated to the control arm is 23.

The total number of participants previously allocated to the interventional arm is 23.

Best wishes,

The Allocation Script

Allocation text file example

001 zz 0 zz 1 zz 1 zz 0 zz 1 zz 0 zz 1 zz 0 zz 0 zz 0 zz 1 zz 0 zz 6 zz 9 zz 2012 zz 62 zz

003 zz 1 zz 0 zz 1 zz 0 zz 0 zz 1 zz 1 zz 0 zz 0 zz 0 zz 0 zz 1 zz 10 zz 9 zz 2012 zz 59 zz

002 zz 1 zz 0 zz 1 zz 0 zz 1 zz 0 zz 1 zz 0 zz 0 zz 0 zz 1 zz 0 zz 17 zz 9 zz 2012 zz 53 zz

004 zz 0 zz 1 zz 1 zz 0 zz 0 zz 1 zz 1 zz 0 zz 0 zz 0 zz 0 zz 1 zz 24 zz 10 zz 2012 zz 34 zz

005 zz 1 zz 0 zz 1 zz 0 zz 1 zz 0 zz 1 zz 0 zz 0 zz 0 zz 0 zz 1 zz 25 zz 10 zz 2012 zz 64 zz

007 zz 1 zz 0 zz 0 zz 1 zz 1 zz 0 zz 1 zz 0 zz 0 zz 0 zz 1 zz 0 zz 28 zz 11 zz 2012 zz 74 zz

008 zz 0 zz 1 zz 0 zz 1 zz 1 zz 0 zz 1 zz 0 zz 0 zz 0 zz 0 zz 1 zz 28 zz 11 zz 2012 zz 72 zz

008 zz 0 zz 1 zz 0 zz 1 zz 1 zz 0 zz 1 zz 0 zz 0 zz 0 zz 1 zz 0 zz 28 zz 11 zz 2012 zz 72 zz

009 zz 0 zz 1 zz 1 zz 0 zz 1 zz 0 zz 1 zz 0 zz 0 zz 0 zz 0 zz 1 zz 5 zz 12 zz 2012 zz 64 zz

009 zz 0 zz 1 zz 1 zz 0 zz 1 zz 0 zz 1 zz 0 zz 0 zz 0 zz 1 zz 0 zz 5 zz 12 zz 2012 zz 64 zz

006 zz 1 zz 0 zz 1 zz 0 zz 1 zz 0 zz 1 zz 0 zz 0 zz 0 zz 0 zz 1 zz 5 zz 12 zz 2012 zz 64 zz

010 zz 0 zz 1 zz 1 zz 0 zz 0 zz 1 zz 1 zz 0 zz 0 zz 0 zz 1 zz 0 zz 8 zz 1 zz 2013 zz 50 zz

012 zz 1 zz 0 zz 0 zz 1 zz 0 zz 1 zz 1 zz 0 zz 0 zz 0 zz 1 zz 0 zz 8 zz 1 zz 2013 zz 70 zz

017 zz 1 zz 0 zz 0 zz 1 zz 1 zz 0 zz 1 zz 0 zz 0 zz 0 zz 0 zz 1 zz 8 zz 1 zz 2013 zz 79 zz

019 zz 0 zz 1 zz 0 zz 1 zz 0 zz 1 zz 1 zz 0 zz 0 zz 0 zz 0 zz 1 zz 8 zz 1 zz 2013 zz 73 zz

015 zz 0 zz 1 zz 1 zz 0 zz 1 zz 0 zz 0 zz 0 zz 0 zz 1 zz 1 zz 0 zz 9 zz 1 zz 2013 zz 45 zz

016 zz 1 zz 0 zz 0 zz 1 zz 1 zz 0 zz 1 zz 0 zz 0 zz 0 zz 1 zz 0 zz 9 zz 1 zz 2013 zz 67 zz

012 zz 0 zz 1 zz 0 zz 1 zz 1 zz 0 zz 1 zz 0 zz 0 zz 0 zz 0 zz 1 zz 9 zz 1 zz 2013 zz 70 zz

013 zz 0 zz 1 zz 1 zz 0 zz 1 zz 0 zz 0 zz 1 zz 0 zz 0 zz 0 zz 1 zz 9 zz 1 zz 2013 zz 53 zz

014 zz 0 zz 1 zz 0 zz 1 zz 1 zz 0 zz 1 zz 0 zz 0 zz 0 zz 1 zz 0 zz 9 zz 1 zz 2013 zz 73 zz

020 zz 0 zz 1 zz 1 zz 0 zz 1 zz 0 zz 1 zz 0 zz 0 zz 0 zz 1 zz 0 zz 14 zz 1 zz 2013 zz 62 zz

018 zz 1 zz 0 zz 0 zz 1 zz 1 zz 0 zz 1 zz 0 zz 0 zz 0 zz 0 zz 1 zz 14 zz 1 zz 2013 zz 68 zz

021 zz 1 zz 0 zz 0 zz 1 zz 1 zz 0 zz 1 zz 0 zz 0 zz 0 zz 1 zz 0 zz 14 zz 1 zz 2013 zz 76 zz

022 zz 1 zz 0 zz 0 zz 1 zz 1 zz 0 zz 0 zz 0 zz 1 zz 0 zz 0 zz 1 zz 14 zz 1 zz 2013 zz 82 zz

023 zz 1 zz 0 zz 0 zz 1 zz 1 zz 0 zz 1 zz 0 zz 0 zz 0 zz 0 zz 1 zz 16 zz 1 zz 2013 zz 71 zz

024 zz 0 zz 1 zz 0 zz 1 zz 1 zz 0 zz 1 zz 0 zz 0 zz 0 zz 1 zz 0 zz 16 zz 1 zz 2013 zz 68 zz

025 zz 1 zz 0 zz 1 zz 0 zz 1 zz 0 zz 1 zz 0 zz 0 zz 0 zz 1 zz 0 zz 16 zz 1 zz 2013 zz 62 zz

027 zz 0 zz 1 zz 1 zz 0 zz 1 zz 0 zz 1 zz 0 zz 0 zz 0 zz 0 zz 1 zz 22 zz 1 zz 2013 zz 40 zz

028 zz 0 zz 1 zz 1 zz 0 zz 1 zz 0 zz 0 zz 1 zz 0 zz 0 zz 0 zz 1 zz 23 zz 1 zz 2013 zz 45 zz

026 zz 0 zz 1 zz 0 zz 1 zz 1 zz 0 zz 1 zz 0 zz 0 zz 0 zz 0 zz 1 zz 23 zz 1 zz 2013 zz 75 zz

029 zz 1 zz 0 zz 1 zz 0 zz 1 zz 0 zz 1 zz 0 zz 0 zz 0 zz 1 zz 0 zz 30 zz 1 zz 2013 zz 62 zz

031 zz 0 zz 1 zz 1 zz 0 zz 1 zz 0 zz 1 zz 0 zz 0 zz 0 zz 1 zz 0 zz 1 zz 2 zz 2013 zz 46 zz

030 zz 0 zz 1 zz 1 zz 0 zz 1 zz 0 zz 1 zz 0 zz 0 zz 0 zz 0 zz 1 zz 4 zz 2 zz 2013 zz 33 zz

032 zz 0 zz 1 zz 0 zz 1 zz 1 zz 0 zz 1 zz 0 zz 0 zz 0 zz 1 zz 0 zz 5 zz 2 zz 2013 zz 67 zz

034 zz 1 zz 0 zz 1 zz 0 zz 1 zz 0 zz 1 zz 0 zz 0 zz 0 zz 0 zz 1 zz 25 zz 2 zz 2013 zz 48 zz

035 zz 0 zz 1 zz 0 zz 1 zz 0 zz 1 zz 1 zz 0 zz 0 zz 0 zz 1 zz 0 zz 27 zz 2 zz 2013 zz 75 zz

036 zz 1 zz 0 zz 0 zz 1 zz 1 zz 0 zz 1 zz 0 zz 0 zz 0 zz 0 zz 1 zz 27 zz 2 zz 2013 zz 75 zz

040 zz 0 zz 1 zz 0 zz 1 zz 0 zz 1 zz 1 zz 0 zz 0 zz 0 zz 0 zz 1 zz 14 zz 3 zz 2013 zz 77 zz

041 zz 1 zz 0 zz 1 zz 0 zz 0 zz 1 zz 1 zz 0 zz 0 zz 0 zz 1 zz 0 zz 20 zz 3 zz 2013 zz 62 zz

041 zz 1 zz 0 zz 1 zz 0 zz 0 zz 1 zz 1 zz 0 zz 0 zz 0 zz 0 zz 1 zz 20 zz 3 zz 2013 zz 62 zz

039 zz 1 zz 0 zz 0 zz 1 zz 0 zz 1 zz 1 zz 0 zz 0 zz 0 zz 1 zz 0 zz 20 zz 3 zz 2013 zz 69 zz

043 zz 1 zz 0 zz 0 zz 1 zz 1 zz 0 zz 1 zz 0 zz 0 zz 0 zz 1 zz 0 zz 20 zz 3 zz 2013 zz 74 zz

044 zz 1 zz 0 zz 1 zz 0 zz 1 zz 0 zz 1 zz 0 zz 0 zz 0 zz 0 zz 1 zz 26 zz 3 zz 2013 zz 48 zz

042 zz 0 zz 1 zz 1 zz 0 zz 1 zz 0 zz 1 zz 0 zz 0 zz 0 zz 1 zz 0 zz 27 zz 3 zz 2013 zz 60 zz

045 zz 1 zz 0 zz 0 zz 1 zz 0 zz 1 zz 1 zz 0 zz 0 zz 0 zz 0 zz 1 zz 3 zz 4 zz 2013 zz 70 zz

046 zz 1 zz 0 zz 1 zz 0 zz 1 zz 0 zz 1 zz 0 zz 0 zz 0 zz 1 zz 0 zz 8 zz 4 zz 2013 zz 56 zz

048 zz 1 zz 0 zz 1 zz 0 zz 1 zz 0 zz 1 zz 0 zz 0 zz 0 zz 0 zz 1 zz 10 zz 4 zz 2013 zz 63 zz

047 zz 1 zz 0 zz 1 zz 0 zz 1 zz 0 zz 1 zz 0 zz 0 zz 0 zz 1 zz 0 zz 10 zz 4 zz 2013 zz 61 zz

049 zz 1 zz 0 zz 0 zz 1 zz 1 zz 0 zz 1 zz 0 zz 0 zz 0 zz 0 zz 1 zz 16 zz 4 zz 2013 zz 73 zz

051 zz 1 zz 0 zz 0 zz 1 zz 1 zz 0 zz 1 zz 0 zz 0 zz 0 zz 1 zz 0 zz 25 zz 4 zz 2013 zz 83 zz

050 zz 1 zz 0 zz 1 zz 0 zz 1 zz 0 zz 1 zz 0 zz 0 zz 0 zz 0 zz 1 zz 1 zz 5 zz 2013 zz 58 zz

054 zz 0 zz 1 zz 0 zz 1 zz 1 zz 0 zz 1 zz 0 zz 0 zz 0 zz 0 zz 1 zz 8 zz 5 zz 2013 zz 75 zz

055 zz 0 zz 1 zz 1 zz 0 zz 1 zz 0 zz 1 zz 0 zz 0 zz 0 zz 1 zz 0 zz 20 zz 5 zz 2013 zz 49 zz

056 zz 1 zz 0 zz 1 zz 0 zz 1 zz 0 zz 1 zz 0 zz 0 zz 0 zz 1 zz 0 zz 22 zz 5 zz 2013 zz 57 zz

057 zz 1 zz 0 zz 1 zz 0 zz 1 zz 0 zz 1 zz 0 zz 0 zz 0 zz 0 zz 1 zz 3 zz 6 zz 2013 zz 25 zz

059 zz 0 zz 1 zz 1 zz 0 zz 1 zz 0 zz 1 zz 0 zz 0 zz 0 zz 0 zz 1 zz 5 zz 6 zz 2013 zz 41 zz

061 zz 0 zz 1 zz 0 zz 1 zz 1 zz 0 zz 1 zz 0 zz 0 zz 0 zz 1 zz 0 zz 12 zz 6 zz 2013 zz 73 zz

062 zz 1 zz 0 zz 1 zz 0 zz 1 zz 0 zz 1 zz 0 zz 0 zz 0 zz 1 zz 0 zz 18 zz 6 zz 2013 zz 30 zz

063 zz 1 zz 0 zz 1 zz 0 zz 1 zz 0 zz 1 zz 0 zz 0 zz 0 zz 0 zz 1 zz 18 zz 6 zz 2013 zz 57 zz

066 zz 0 zz 1 zz 0 zz 1 zz 0 zz 1 zz 1 zz 0 zz 0 zz 0 zz 0 zz 1 zz 19 zz 6 zz 2013 zz 72 zz

064 zz 1 zz 0 zz 1 zz 0 zz 0 zz 1 zz 1 zz 0 zz 0 zz 0 zz 1 zz 0 zz 24 zz 6 zz 2013 zz 48 zz

065 zz 1 zz 0 zz 0 zz 1 zz 1 zz 0 zz 1 zz 0 zz 0 zz 0 zz 1 zz 0 zz 24 zz 6 zz 2013 zz 75 zz

068 zz 0 zz 1 zz 1 zz 0 zz 1 zz 0 zz 1 zz 0 zz 0 zz 0 zz 0 zz 1 zz 25 zz 6 zz 2013 zz 44 zz

067 zz 0 zz 1 zz 1 zz 0 zz 1 zz 0 zz 1 zz 0 zz 0 zz 0 zz 1 zz 0 zz 27 zz 6 zz 2013 zz 63 zz

070 zz 0 zz 1 zz 1 zz 0 zz 0 zz 1 zz 1 zz 0 zz 0 zz 0 zz 0 zz 1 zz 8 zz 7 zz 2013 zz 26 zz

069 zz 1 zz 0 zz 1 zz 0 zz 1 zz 0 zz 1 zz 0 zz 0 zz 0 zz 0 zz 1 zz 15 zz 7 zz 2013 zz 54 zz

073 zz 0 zz 1 zz 0 zz 1 zz 1 zz 0 zz 1 zz 0 zz 0 zz 0 zz 1 zz 0 zz 15 zz 7 zz 2013 zz 69 zz

074 zz 0 zz 1 zz 0 zz 1 zz 1 zz 0 zz 1 zz 0 zz 0 zz 0 zz 0 zz 1 zz 15 zz 7 zz 2013 zz 67 zz

071 zz 1 zz 0 zz 1 zz 0 zz 1 zz 0 zz 1 zz 0 zz 0 zz 0 zz 1 zz 0 zz 18 zz 7 zz 2013 zz 44 zz

075 zz 1 zz 0 zz 1 zz 0 zz 1 zz 0 zz 1 zz 0 zz 0 zz 0 zz 0 zz 1 zz 22 zz 7 zz 2013 zz 53 zz

076 zz 1 zz 0 zz 0 zz 1 zz 1 zz 0 zz 1 zz 0 zz 0 zz 0 zz 1 zz 0 zz 1 zz 8 zz 2013 zz 67 zz

085 zz 0 zz 1 zz 0 zz 1 zz 1 zz 0 zz 1 zz 0 zz 0 zz 0 zz 0 zz 1 zz 28 zz 8 zz 2013 zz 79 zz

082 zz 0 zz 1 zz 1 zz 0 zz 1 zz 0 zz 1 zz 0 zz 0 zz 0 zz 1 zz 0 zz 28 zz 8 zz 2013 zz 64 zz

079 zz 0 zz 1 zz 1 zz 0 zz 1 zz 0 zz 1 zz 0 zz 0 zz 0 zz 0 zz 1 zz 28 zz 8 zz 2013 zz 46 zz

078 zz 1 zz 0 zz 1 zz 0 zz 1 zz 0 zz 1 zz 0 zz 0 zz 0 zz 1 zz 0 zz 29 zz 8 zz 2013 zz 64 zz

084 zz 1 zz 0 zz 0 zz 1 zz 1 zz 0 zz 0 zz 0 zz 1 zz 0 zz 0 zz 1 zz 2 zz 9 zz 2013 zz 66 zz

086 zz 0 zz 1 zz 1 zz 0 zz 0 zz 1 zz 1 zz 0 zz 0 zz 0 zz 1 zz 0 zz 9 zz 9 zz 2013 zz 41 zz

087 zz 1 zz 0 zz 1 zz 0 zz 1 zz 0 zz 1 zz 0 zz 0 zz 0 zz 0 zz 1 zz 11 zz 9 zz 2013 zz 62 zz

083 zz 1 zz 0 zz 1 zz 0 zz 1 zz 0 zz 1 zz 0 zz 0 zz 0 zz 1 zz 0 zz 16 zz 9 zz 2013 zz 63 zz

HTML code for Web Page 1.

<HTML>

<h1>Allocation</h1>

<p>&nbsp;</p>

<strong>Form to allocate a participant </strong><form action="http://users.ox.ac.uk/CGI-bin/safePerl/clme0164/temp1allocation.CGI" method="post">

<h1><span style="font-family: Arial, Helvetica, sans-serif; color: #330066; font-size: small;"><input name="fromwho" type="hidden" value="chris.ocallaghan@ndm.ox.ac.uk" /> <input name="replytowho" type="hidden" value="chris.ocallaghan@ndm.ox.ac.uk" /> <input name="replyfromwho" type="hidden" value="chris.ocallaghan@ndm.ox.ac.uk" /> <input name="replywhatsubject" type="hidden" value="replywhatsubjecttext" /> <input name="replywhatcontents" type="hidden" value="replywhatcontentstext" /> <input name="source" type="hidden" value="booked" /> <input name="sendformto" type="hidden" value="name@domain.ac.uk" /> </span></h1>

<p>If you wish to perform an allocation complete all parts of this form. <br />You must complete a separate form for each allocation.</p>

<p>Your email address: <input name="whatsubmitteremail" size="35" type="text" /></p>

<p>Participant id: <input name="whatparticipantid" type="text" /></p>

<p>Age: <input name="whatage" type="text" /></p>

<p>Gender :<select name="whatgender">

<option selected="selected" value="male">Male</option>

<option value="female">Female</option>

</select></p>

<p>Ethnicity:<select name="whatethnicity">

<option selected="selected" value="white">White: British - code A</option>

<option value="white">White: Irish - code B</option>

<option value="white">White: Any other White background - code C</option>

<option value="black">Mixed: White and Black Caribbean - code D</option>

<option value="black">Mixed: White and Black African - code E</option>

<option value="asian">Mixed: White and Asian - code F</option>

<option value="white">Mixed: Any other mixed background - code G</option>

<option value="asian">Asian or Asian British: Indian - code H</option>

<option value="asian">Asian or Asian British: Pakistani - code J</option>

<option value="asian">Asian or Asian British: Bangladeshi - code K</option>

<option value="asian">Asian or Asian British: Any other Asian background - code L</option>

<option value="black">Black or Black British: Caribbean - code M</option>

<option value="black">Black or Black British: African - code N</option>

<option value="black">Black or Black British: Any other Black background - code P</option>

<option value="chinese">Other Ethnic Groups: Chinese - code R</option>

<option value="white">Other Ethnic Groups: Any other ethnic group - code S</option>

<option value="white">Not stated - code Z</option>

</select></p>

<p>Diabetic:<select name="whatdiabetic">

<option selected="selected" value="nondiabetic">Non-diabetic</option>

<option value="diabetic">Diabetic</option>

</select></p>

<p><input name="Reset" type="reset" value="Clear Form" /> <input name="Submit" type="submit" value="Submit Application" /></p>

</form>

</HTML>
